# A deep reinforcement learning approach for dynamic transaction fee adjustment in Ethereum

**DOI:** 10.1038/s41598-026-46368-2

**Published:** 2026-03-31

**Authors:** Huisu Jang, Jaewoong Shim

**Affiliations:** 1 School of Finance, Soongsil University, 369 Sangdo-ro,Dongjak-gu, 06978 Seoul, Republic of Korea; 2 Department of Industrial Engineering, Seoul National University of Science and Technology, 232 Gongneung-ro,Nowon-gu, 01811 Seoul, Republic of Korea

**Keywords:** Ethereum, Deep reinforcement learning, Blockchain, Transaction fee markets, Engineering, Mathematics and computing, Physics

## Abstract

Blockchain users pay transaction fees to miners or block proposers who validate and add transactions to the distributed ledger. Ethereum introduces the concept of gas to decouple transaction costs from Ether’s price volatility, calculating fees based on gas units. The current mechanism defined by Ethereum Improvement Proposal (EIP) 1559 dynamically adjusts the base fee according to block gas usage. However, its rule-based adjustment can lead to unstable gas consumption when demand fluctuates within a narrow range and struggles to respond efficiently to sudden demand spikes, such as during non-fungible token (NFT) drops. To address these limitations, we propose a deep reinforcement learning-based transaction fee mechanism that learns an adaptive base-fee update policy. Our approach maintains gas consumption close to the target level across various demand scenarios and stabilizes transaction fees and gas usage per block even under abrupt demand shifts. These results demonstrate that the proposed method provides a more adaptive and resilient fee adjustment mechanism compared to the current EIP-1559 model.

## Introduction

Transaction fee mechanisms are critical components of blockchain networks, directly impacting user experience, system efficiency, and security. In platforms like Ethereum, where value transfers and smart contract executions occur frequently, an effective fee mechanism ensures smooth transaction executions for users and fair compensation for miners (the term *miner* refers to block proposers, i.e., validators, in Ethereum’s Proof-of-Stake system). Designing an optimal fee mechanism is essential to balance these interests, prevent network congestion, and maintain blockchain integrity^1,2^.

Ethereum Improvement Proposal 1559 (EIP-1559) was introduced to address inefficiencies in the original first-price auction model for transaction fees. Implemented during the London hard fork^3^, EIP-1559 aims to improve fee predictability and user experience by introducing a dynamically adjusting base fee and an optional priority fee (tip)^4^. The base fee adjusts according to network demand, increasing when blocks are over 50% full and decreasing when they are underutilized.

Despite these advancements, EIP-1559 exhibits limitations under extreme demand conditions. Moreover, EIP-1559 struggles to handle sudden demand spikes effectively, such as during non-fungible token (NFT) drops or other high-demand events^5^. In these periods, the base fee adjustment may not respond swiftly enough, resulting in network congestion and elevated transaction fees.

**Fig. 1:**
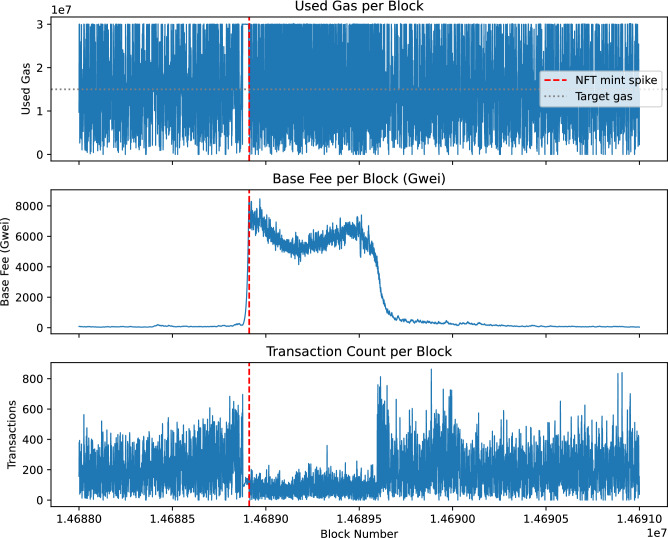
Gas used per block and base fee during the demand spike triggered by the Otherdeed NFT mint in May 2022 (block range 14,688,000–14,691,000). Gas used per block: mean = 15,415,347, variance = $1.08 \times 10^{14}$; Base fee (Gwei): mean = 1536.15, variance = $6.06 \times 10^{6}$; Transaction count mean = 183.95, variance = $2.15 \times 10^4$; Gas utilization mean = 0.5138, variance = $1.20 \times 10^1$.

Figure 1 illustrates gas usage and base fee dynamics during the demand spike triggered by the Otherdeed NFT mint in May 2022 (block range 14,688,000–14,691,000). During this period, gas usage per block remains close to the block gas limit, while the base fee increases sharply in response to the sudden surge in transaction demand. The data show substantial variability in both gas usage and base fee levels (gas used mean = 15,415,347, variance = $1.08 \times 10^{14}$; base fee mean = 1536.15 Gwei, variance = $6.06 \times 10^{6}$; Transaction count mean = 183.95, variance = $2.15 \times 10^4$; Gas utilization mean = 0.5138, variance = $1.20 \times 10^1$). We use transaction count and gas utilization (gas used relative to the block gas limit) as indicators of network congestion.

**Our Contributions: **In this paper, we propose a novel transaction fee mechanism using deep reinforcement learning (DRL). While EIP-1559 adjusts gas fees solely based on gas usage, our DRL-based approach allows for the incorporation of additional variables-such as mempool information before and after block creation-enabling the model to better capture demand dynamics and respond more flexibly. To the best of our knowledge, this is the first attempt to leverage DRL for optimizing transaction fee adjustments in a blockchain context. Our DRL-based mechanism outperforms EIP-1559 in several key aspects, as demonstrated through extensive simulations. Unlike prior studies that analyze the dynamics of EIP-1559 or propose rule-based modifications to the base-fee mechanism, we formulate the fee adjustment problem as a learning-based control problem and learn the update policy directly through reinforcement learning.

First, our method achieves lower variance in both gas used per block and the base fee, leading to more stable and predictable transaction fees. The reinforcement learning agent learns to adjust the base fee with greater precision, preventing the large swings observed under EIP-1559. Second, we maintain the average gas used per block close to the target gas limit, ensuring efficient utilization of block space without overloading the network. Third, our mechanism adapts effectively to sudden changes in block demand, mitigating the impact of demand spikes on transaction costs and network performance.

The remainder of this paper is organized as follows: Section 2 covers background. Section 3 reviews related work. In Section 4, we introduce our proposed approach, including the formulation of the reinforcement learning model. Section 5 describes our experimental design. Section 6 discusses the experimental results. Finally, Section 7 concludes the paper and suggests directions for future research.

## Background

### Transaction fee mechanism

In Ethereum, users pay transaction fees denominated in *gas*, which represents the computational effort required to execute operations on the blockchain^6^. Each instruction in the Ethereum Virtual Machine (EVM) consumes a fixed amount of gas, and the total transaction fee is determined by the gas consumed and the gas price.

Prior to EIP-1559, Ethereum employed a first-price auction mechanism in which users specified the gas price they were willing to pay per unit of gas. Miners prioritized transactions with higher gas prices to maximize their revenue. Although simple, this mechanism often resulted in inefficient bidding behavior during periods of network congestion, leading to highly volatile and unpredictable transaction fees^7^. Users frequently struggled to estimate an appropriate gas price, which could result in either excessive fees or delayed transaction confirmation.

To address these issues, Ethereum introduced EIP-1559 in August 2021 as part of the London hard fork. This mechanism introduces a dynamically adjusting base fee ($b_t$), representing the minimum gas price required for inclusion in the *t*-th block. The base fee is updated based on network demand. If the gas used in block *t* ($y_t$) exceeds the target gas ($y_{\text {target}}$), the base fee increases; otherwise, it decreases. The update rule is defined as

1 $$\begin{aligned} b_{t+1} = b_t \times \left( 1 + d \times \frac{y_t - y_{\text {target}}}{y_{\text {target}}}\right) \end{aligned}$$

where $y_{\text {target}}$ denotes the target gas per block (e.g., 15 million gas) and *d* represents the maximum adjustment rate of the base fee (typically capped at 12.5%).

Users submitting transactions specify two parameters for transaction *i*:

- **Max fee per gas **($f^{i}_{\text {max}}$): The maximum amount they are willing to pay per unit of gas.
- **Max priority fee per gas **($f^{i}_{\text {tip}}$): The maximum extra fee they are willing to pay to miners or block proposers for priority inclusion.

Transactions submitted by users are transmitted to the mempool (memory pool) through the peer-to-peer network. Miners then select transactions that maximize their profits from the mempool, include them in candidate blocks, and begin the mining process. If a miner succeeds in mining a block, they receive the total transaction fees minus the burned base fee.

The actual fee per unit of gas that transaction *i* pays is calculated as $\min (f^{i}_{\text {max}},\ b_t + f^{i}_{\text {tip}})$, and the total transaction cost is this amount multiplied by the gas consumed by the transaction. The miner’s reward per unit of gas is $\min (f^{i}_{\text {max}},\ b_t + f^{i}_{\text {tip}}) - b_t$, as the base fee is burned.

Currently, the utility users derive from placing transactions on the blockchain can be represented by the max fee ($f^{i}_{\text {max}}$) they are willing to pay, reflecting their valuation of the transaction - a concept consistent with existing studies^8^. EIP-1559 is designed to offer users more efficient and predictable fee options by utilizing a dynamically adjusting base fee and an optional priority fee. This mechanism aims to enhance fee stability and user experience by responding adaptively to network congestion levels. The theoretical and experimental implications of EIP-1559 have been studied extensively, and we will explore these studies further in the related work section.

### Deep reinforcement learning

Reinforcement Learning (RL) is a machine learning paradigm where an agent learns to make decisions by interacting with an environment to achieve a specific goal^9^. RL is typically formulated as a *Markov Decision Process* (MDP), which provides a mathematical framework consisting of states, actions, transition probabilities, and rewards. In an MDP, the agent aims to learn a policy that maximizes the cumulative reward by making optimal decisions in each state.

Deep Reinforcement Learning (DRL) extends RL by integrating deep neural networks to handle high-dimensional and continuous state and action spaces^10^. This combination allows agents to generalize from experiences and make complex decisions, making DRL suitable for a wide range of applications, including dynamic pricing^11,12^, scheduling^13^, network routing^14^, portfolio management^15^, autonomous driving^16^, and robot control^17^. The application of DRL in blockchain systems is relatively unexplored, with only a few studies applying it to blockchain systems^18–21^.

## Related work

Several theoretical and empirical studies have analyzed the impact of EIP-1559 on the Ethereum network. Roughgarden^8^ confirmed the theoretical soundness of EIP-1559 through game-theoretic analysis, demonstrating its effectiveness in reducing inflation via base fee burning and minimizing fee dispersion with variable block sizes. An alternative fee mechanism with incentive compatibility guarantees was also proposed^22^. However, these studies highlighted a persistent challenge: fee estimation during periods of rapid demand fluctuations remains difficult, consistent with empirical findings.

Liu et al.^4^ provided an in-depth examination by comparing data from periods before and after the London hard fork. The findings indicated that EIP-1559 reduced the volatility of gas prices within blocks and decreased transaction waiting times, improving user experience. However, despite these improvements, EIP-1559 had minimal impact on the absolute level of gas fees and consensus security. Additionally, increased volatility in the ETH price led to longer user waiting times and a higher frequency of large blocks exceeding the target size. These results suggest that while EIP-1559 addresses certain market needs, further refinements are necessary to fully optimize the fee mechanism.

Leonardos et al.^5^ provided bounds on the change rate of the base fee adjustment to ensure convergence, utilizing concepts from game theory and dynamical systems. They explored scenarios where the base fee dynamics fail to converge, leading to chaotic behavior characterized by the gas per block alternating between maximum and minimum levels. Specifically, they highlighted that when users’ valuations of transactions are distributed within a narrow range with minimal differences, the fee dynamics may fail to converge and enter a state of chaos. In a subsequent study, the authors further examined the dynamics of transaction fees and gas used per block under EIP-1559^23^. They demonstrated that despite potential chaotic situations, the time-average gas used per block under EIP-1559 possesses a lower bound equal to the block target and an upper bound exceeding the target by approximately 6%. When users’ valuations are narrowly distributed, small changes in the base fee can cause the block size to oscillate between maximum capacity and very small sizes, leading to inefficient utilization of block space.

Recent work has begun to treat blockchain economic variables as data-driven control problems^24,25^. Hu et al.^25^ propose a transaction-based classification and detection approach to identify and classify Ethereum smart contracts based on transaction behaviors by employing LSTM networks. Wang et al.^24^ propose a temporal graph attention network approach for detecting phishing scams in Ethereum. They construct temporal graph embeddings considering the transaction topology, node features, and edge features dynamically over time. DRL is employed to characterize selfish mining strategies and quantify how miner bribery lowers Bitcoin’s security threshold^26^. Reinforcement learning has also been applied to joint transaction fee selection and scheduling in IoT-blockchain systems, showing clear throughput gains over Q-learning baselines^27^. However, all of these studies focus on fee estimation or security analysis, not on adapting the fee adjustment mechanism itself. In contrast, our work treats the base-fee adjustment as a policy learning problem and optimizes the update rule using deep reinforcement learning.

Our work aligns with the objectives of Reijsbergen et al.^28^, who aimed to create better market conditions by varying the change rate of the base fee. However, while they adjusted the change rate using a rule-based update mechanism, our model leverages DRL to enable more nuanced and adaptive control of the change rate. This allows the mechanism to respond more effectively to real-time network conditions, minimizing the risk of chaotic fee dynamics and improving overall network performance.

While several blockchain studies have explored improvements in cryptographic protocols or payment models, such as aggregate signature schemes and blockchain-based virtual payment systems, these works primarily focus on security, scalability, or payment execution rather than transaction fee adjustment mechanisms. In contrast, our work addresses the problem of dynamically adjusting the base-fee update rule itself. To the best of our knowledge, no prior work tunes the EIP-1559 base-fee change rate using learning-based control, leaving an important gap that our reinforcement-learning model aims to address.

## Proposed method

Unlike the rule-based update in EIP-1559, which adjusts the base fee solely based on gas usage in the previous block, real Ethereum fee dynamics depend on multiple interacting factors, including mempool conditions, transaction arrival patterns, and user bidding behavior. These factors jointly influence transaction inclusion and network congestion, making it difficult to design effective adaptive rules using simple heuristics. A learning-based approach provides a natural way to capture these complex relationships by directly learning a state-dependent adjustment policy from system interactions. Therefore, we employ DRL to learn an adaptive base-fee adjustment mechanism that can respond to varying network conditions.

**Fig. 2:**
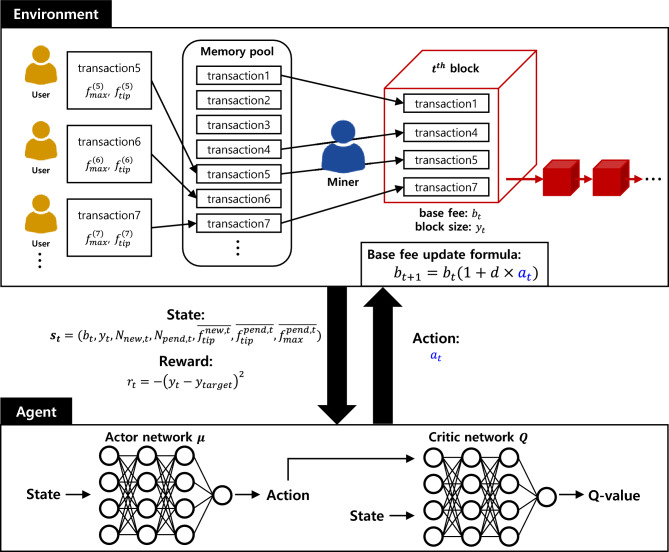
Overview of the proposed DRL-based fee adjustment mechanism. At each block *t*, the agent observes the state $\textbf{s}_t$, which includes base fee, gas usage, and mempool information. The actor network outputs an action $a_t$, which determines the base fee adjustment for the next block. The environment then generates the next state and reward based on the resulting gas usage, and the critic network evaluates the expected cumulative reward during training.

An overview of the proposed method is presented in Figure 2. In the environment, the base fee for the next block is determined based on the action provided by the agent, while the gas used per block is influenced by the decisions of both users and miners. The reward is then calculated according to how close the gas used per block is to the target gas used. The agent derives the optimal action based on the state and reward information provided by the environment. The agent is composed of an actor network and a critic network: the actor network takes the state as input and outputs an action, while the critic network takes both the action generated by the actor network and the state as inputs, and outputs the Q-value, representing the expected cumulative future reward. We employed the Deep Deterministic Policy Gradient (DDPG) method^29^ for this purpose. DDPG is particularly suited for handling continuous action spaces, utilizing both actor and critic models.

### Formulation as a reinforcement learning problem

To apply DRL to the transaction fee adjustment mechanism, we model the problem as a MDP, which consists of states, actions, rewards, and state transitions. Here, *t* represents the index of the *t*-th block, which increments by 1 with the generation of each new block.

**State **($\textbf{s}_t$): For the state, we leverage two pieces of block information (base fee and gas used), which were also used in the original EIP-1559 formula, along with five additional pieces of information about the mempool status. More specifically, the state at the *t*-th block $\textbf{s}_t$ is a vector includes the following seven elements:

- $b_{t}$: Base fee per gas in the *t*-th block.
- $y_t$: Gas used in the *t*-th block.
- $N_{new,t}$: Number of newly arrived transactions in the mempool after the $(t-1)$-th block was generated.
- $N_{pend,t}$: Number of pending transactions in the mempool after the *t*-th block was generated.
- $\overline{f^{new,t}_{tip}}$: Average priority fee of the newly arrived transactions.
- $\overline{f^{pend,t}_{tip}}$: Average priority fee of the pending transactions.
- $\overline{f^{pend,t}_{max}}$: Average max fee of the pending transactions.

**Action **($a_t$): Given the state $\textbf{s}t$, the agent determines the action $a_t$. Instead of directly defining the action $a_t$ as the next base fee $b{t+1}$, we align it with Equation 1 of EIP-1559 to promote greater stability in the agent’s behavior. The action $a_t$ is restricted to a scalar value between −1 and 1 and is used to determine the next base fee $b_{t+1}$ through the following formula:

2 $$\begin{aligned} b_{t+1} = b_t(1 + d \times a_{t}) \end{aligned}$$

This formulation is inspired by the EIP-1559, with the adjustment determined by the agent’s action. The hyperparameter *d*, representing the maximum change rate of base fee, controls the extent to which our DRL-based method can influence the base fee dynamics.

**Reward **($r_t$): The reward is designed to encourage the gas used for the *t*-th block to be as close as possible to the target gas value. The reward function is defined as:

3 $$\begin{aligned} r_t = -(y_{t} - y_{target})^2 \end{aligned}$$

This quadratic form ensures that the reward decreases as the gas used deviates from the target. This reward formulation directly aligns with the primary objective of EIP-1559, which is to maintain gas usage close to the target level. Stabilizing gas usage around the target indirectly contributes to fee stability, as large deviations in block utilization tend to cause abrupt base fee adjustments.

**Transition:** Once the base fee is determined based on the agent’s action, the next state is generated according to the behavior of users and miners, following the dynamics described in the Background section.

### Training procedure

In our problem, each interaction between the agent and the environment corresponds to the generation of a single block. The environment dynamics between blocks, including transaction arrivals and mempool updates, are modeled as described in the Simulation Environment section. Since blocks are continuously generated, this forms an infinite-horizon MDP without clear episode boundaries. For practical training purposes, we define an episode as the completion of $T = 1{,}000$ blocks. At the start of each episode, the transaction arrival process is randomly set to allow the model to learn under various conditions, and the mempool is reset to an empty state.

During training, the agent collects experiences $(\textbf{s}_t, a_{t}, r_{t+1}, \textbf{s}_{t+1})$ and stores them in a replay buffer $\mathcal {D}$. The networks are updated using minibatches sampled from $\mathcal {D}$.

The actor network $\mu (\textbf{s}_t\,|\,\theta ^\mu )$, parameterized by $\theta ^\mu$, takes the current state $\textbf{s}_t$ as input and outputs an action $a_{t}$. To encourage exploration, Gaussian noise $n_t \sim \mathcal {N}(0, \sigma ^2)$ is added to the action during training:

$$\begin{aligned} a_{t} = \mu (\textbf{s}_t\,|\,\theta ^\mu ) + n_t. \end{aligned}$$

The critic network $Q(\textbf{s}_t, a_{t}\,|\,\theta ^Q)$, parameterized by $\theta ^Q$, estimates the Q-value which represents the expected cumulative future reward. The critic is trained to minimize the loss:

$$\begin{aligned} L(\theta ^Q) = \frac{1}{N} \sum _{i=1}^N \left( q_i - Q(\textbf{s}_i, a_{i}|\theta ^Q) \right) ^2, \end{aligned}$$

where *N* is the minibatch size, and the target Q-value $q_i$ is computed using the target networks:

$$\begin{aligned} q_i = r_{i+1} + \gamma Q'\left( \textbf{s}_{i+1}, \mu '\left( \textbf{s}_{i+1}|\theta ^{\mu '} \right) \big | \theta ^{Q'} \right) . \end{aligned}$$

In this equation, $\gamma \in [0,1)$ is the discount factor, $Q'$ and $\mu '$ are the target networks for the critic and actor, respectively, with parameters $\theta ^{Q'}$ and $\theta ^{\mu '}$. The target networks are soft copies of the original networks and are used to stabilize training by providing consistent targets.

To maximize the expected Q-value, the actor network is updated using the policy gradient^30^ derived from the critic network:

$$\begin{aligned} \nabla _{\theta ^\mu } J \approx \frac{1}{N} \sum _{i=1}^N \nabla _a Q\left( \textbf{s}_i, a\,|\,\theta ^Q \right) \bigg |_{a = \mu (\textbf{s}_i)} \nabla _{\theta ^\mu } \mu (\textbf{s}_i\,|\,\theta ^\mu ). \end{aligned}$$

The target networks are updated using soft updates:

$$\begin{aligned} \theta ^{Q'} \leftarrow \tau \theta ^Q + (1 - \tau ) \theta ^{Q'}, \quad \theta ^{\mu '} \leftarrow \tau \theta ^\mu + (1 - \tau ) \theta ^{\mu '}. \end{aligned}$$

where $\tau$ is the soft update rate, ensuring that the target networks slowly track the learned networks.

The overall training procedure is summarized in Algorithm 1.

**Algorithm 1:**
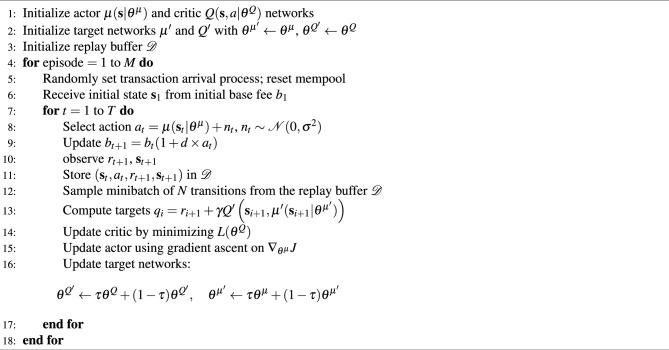
Training Procedure.

### Operating procedure

After completing the training procedure, only the actor network is utilized in operation, as the critic network primarily serves to train the actor during learning. Additionally, the Gaussian noise $n_t$ used for exploration during training is no longer applied to the actor’s output. At each time step, the agent observes the current state $\textbf{s}_t$ and outputs the action $a_t$, which determines the base fee adjustment for the next block. Since the inference required for each block involves only a lightweight forward pass of the actor network, the additional computational overhead is minimal. This enables the fee adjustment mechanism to dynamically adapt to changing network conditions.

## Experimental design

### Simulation environment

To evaluate the performance of our proposed DRL fee adjustment mechanism, we construct a simulation environment that mirrors the assumptions and settings used in existing literature^5,28^. Our simulation models the essential components of the Ethereum network, including blockchain dynamics, user and miner behavior, the transaction demand process (including sudden demand spikes), and the mempool state.

**Blockchain Dynamics:** The blockchain grows by generating blocks sequentially at regular intervals. To focus on the fee mechanism, we simplify the environment by excluding scenarios such as forks or attacks. We assume block generation is deterministic, with each block representing a single iteration where the base fee is updated. We use the gas target of 15,000,000 gas units and a maximum gas limit of 30,000,000 gas units, allowing blocks to expand up to twice the target size as specified in EIP-1559.

We exclude network latency and assume concurrency among all users and miners. All participants have access to a single shared mempool and can access the same list of pending transactions. They receive information about newly generated blocks instantaneously, enabling immediate updates to the base fee.

**User Behavior:** We consider two types of users: strategic and non-strategic. Both submit transactions with a valuation *v* drawn from a distribution *F*. All users set their max fee equal to their transaction valuation, consistent with prior studies^5^, assuming the max fee represents the transaction’s value.

Non-strategic users always submit the same priority fee *e*, the minimum reward required for miners to include their transaction. This compensates miners for the delay caused by adding more transactions, which can reduce their chances of earning a block reward^31^. Strategic users adjust their max priority fee based on the gas used in the previous block. If the previous block’s gas used is below 90% of the limit, they submit *e* like non-strategic users. If it exceeds 90%, they add a random value $\delta$, drawn from distribution *G*, to the median max priority fee of transactions in the previous block. This behavior increases the likelihood of their transactions being included in the next block. We assume all transactions consume 75, 000 gas, and the valuation follows uniform distributions in ranges (80, 90) Gwei and (10, 100) Gwei. This assumption is also consistent with empirical observations that many non-strategic Ethereum users tend to submit transactions with the minimum priority fee.

**Miner Behavior: **We model the block generation process in a PoS system. Each miner (block proposer) is randomly selected per slot and generates one block without considering forks or re-org. We assume miners are myopic, prioritizing transactions by fee to maximize their tip and excluding the maximal extractable value (MEV) from consideration. While MEV influences miner incentives in the current Ethereum system, we exclude it here to focus on the transaction fee mechanism.

**Transaction Arrival Process:** Blocks are generated at regular intervals, each representing one unit of time. Transactions arrive according to a Poisson process with a rate parameter $\lambda _t$ during the interval between the $(t-1)$-th and *t*-th blocks, and the arriving transactions are added to the mempool before the next block is generated. To account for changing demand, we model $\lambda _t$ using a modified compound Poisson process, where the size of each jump follows a normal distribution:

4 $$\begin{aligned} \lambda _t = \lambda _0 + \sum _{i=1}^{N(t)} \max (\lambda _{\text {lower}}, \lambda _{i-1} + Z_i) \end{aligned}$$

- $\lambda _0$: Initial value of $\lambda$ (280).
- *N*(*t*): Number of Poisson events before time *t*, with events following a Poisson rate of 0.01.
- $\lambda _{\text {lower}}$: Lower bound of $\lambda$ (default is 200).
- $Z_i \sim N(0, 50)$: Random change in value at each event time, representing increases or decreases in demand.

**Mempool:** Transactions are stored in a public mempool before inclusion in a block. We consider only the public mempool and assume a single, unified pool for simplicity. Each transaction remains in the mempool for up to 10 blocks; if not included within this time, it is removed.

### Implementation details

The actor and critic networks are both composed of two hidden layers with 256 neurons each, using ReLU activation functions. For the output layers, the actor network uses the hyperbolic tangent activation function, while the critic network uses the ReLU activation function. We performed layer normalization^32^ for all layers in both networks to stabilize training. The Adam optimizer^33^ was employed for training, with learning rates set to $1 \times 10^{-4}$ for the actor and $1 \times 10^{-3}$ for the critic. During operation, only a single forward pass of the relatively simple actor network is required per block. Given that Ethereum blocks are produced approximately every 12 seconds, the resulting computational overhead is negligible and unlikely to affect block propagation or validator performance.

To stabilize the initial training process, actions were determined by random sampling from a uniform distribution on $(-0.25,0.25)$ until 2,000 transitions were stored in the replay buffer. Thereafter, neural network training commenced. To prevent the model from learning undesirable behaviors-such as the base fee converging to zero when block sizes consistently reach the gas limit, or continuously increasing when block sizes approach zero-an episode was terminated if 20 consecutive blocks had a gas usage of either zero or 30 million (the gas limit).

The hyperparameters used in our experiments are summarized in Table 1.

**Table 1:** Summary of hyperparameters.

**Hyperparameter**	**Value**
Maximum change rate of base fee (*d*)	0.2
Discount factor ($\gamma$)	0.9
Soft update rate ($\tau$)	0.005
Replay buffer size	$1 \times 10^{5}$
Minibatch size (*N*)	256
Exploration noise ($\sigma$)	0.05

### Evaluation protocol

To demonstrate the adaptability of our DRL-based approach under changing transaction arrival distributions, we consider five distinct scenarios in which the Poisson rate $\lambda _t$ varies:

- **Scenario 1:** $\lambda _t$ fluctuates within a range that does not exceed the block gas limit.
- **Scenario 2: **$\lambda _t$ gradually increases.
- **Scenario 3: **$\lambda _t$ gradually decreases.
- **Scenario 4: **$\lambda _t$ experiences a temporary sharp peak.
- **Scenario 5: **$\lambda _t$ experiences a temporary sharp drop.

**Fig. 3:**
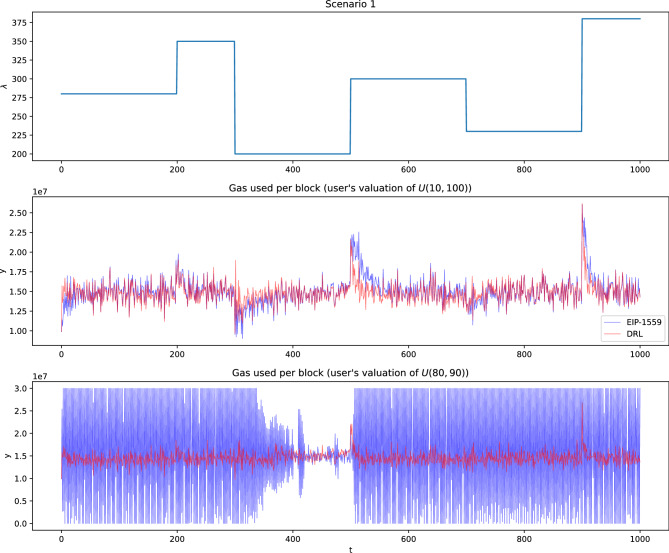
Gas used per block in scenario 1 with strategic users and valuation distributions of *U*(10, 100) and *U*(80, 90).

We generate time series of 1,000 blocks for each scenario. The realized scenarios are illustrated in Figure 3 and Supplementary materials.

We use the average ($\mu _y$) and standard deviation ($\sigma _y$) of the gas used per block, along with the rolling window standard deviation of the base fee ($\sigma _{b,\text {rolling}}$), as performance evaluation metrics. The rolling window standard deviation of the base fee is calculated using a 100-block window, rolling it by one block, and then averaging the resulting values.

## Experimental results

### Main results

Table 2 compares our DRL-based fee adjustment method with EIP-1559 across various scenarios and user behaviors. The results demonstrate that our method achieves lower variance in both gas used per block and base fee, while maintaining the average gas used close to the target gas. To evaluate the robustness of the proposed mechanism under different market conditions, we consider both wide and narrow valuation distributions in our experiments. The narrow valuation range is particularly important because prior studies have shown that EIP-1559 may exhibit unstable dynamics when users’ valuations are concentrated within a small range.

**Table 2:** Performance comparison between EIP-1559 and the proposed DRL-based fee adjustment mechanism across different user types, valuation distributions, and scenarios.

			$\mu _y$ ($\times 10^7$)		$\sigma _y$ ($\times 10^6$)		$\sigma _{b,rolling}$	
User	Valuation	Scenario	EIP-1559	DRL	EIP-1559	DRL	EIP-1559	DRL
Strategic	*U*(10, 100)	1	**1.500**	1.485	1.748	**1.358**	**4.910**	5.226
		2	1.504	**1.503**	1.487	**1.253**	1.939	**1.770**
		3	1.480	**1.484**	1.170	**1.157**	**1.162**	1.338
		4	**1.497**	1.473	1.850	**1.626**	2.180	**2.048**
		5	**1.503**	1.509	1.612	**1.509**	1.771	**1.731**
		avg	**1.497**	1.491	1.573	**1.381**	**2.392**	2.423
	*U*(80, 90)	1	1.572	**1.452**	13.084	**1.415**	4.927	**0.646**
		2	1.585	**1.515**	14.175	**1.442**	5.671	**0.187**
		3	**1.569**	1.428	13.007	**1.306**	4.880	**0.233**
		4	1.585	**1.419**	14.204	**1.488**	5.430	**0.309**
		5	**1.585**	1.589	14.290	**1.415**	5.819	**0.137**
		avg	1.579	**1.481**	13.752	**1.413**	5.345	**0.302**
	avg		1.538	**1.486**	7.663	**1.397**	3.717	**1.189**
Non-Strategic	*U*(10, 100)	1	**1.500**	1.511	1.748	**1.472**	**4.910**	5.589
		2	**1.504**	1.478	1.487	**1.239**	1.939	**1.681**
		3	1.480	**1.497**	**1.170**	1.289	**1.162**	1.618
		4	**1.497**	1.458	1.844	**1.726**	2.181	**2.061**
		5	**1.503**	1.477	1.612	**1.326**	1.771	**1.416**
		avg	**1.497**	1.484	1.572	**1.410**	**2.392**	2.473
	*U*(80, 90)	1	1.573	**1.470**	13.264	**1.427**	5.117	**1.637**
		2	1.589	**1.455**	14.496	**1.318**	6.019	**0.202**
		3	1.572	**1.485**	13.222	**1.343**	5.089	**0.717**
		4	1.584	**1.431**	14.091	**1.828**	5.535	**0.359**
		5	1.589	**1.460**	14.541	**1.384**	6.133	**0.198**
		avg	1.581	**1.460**	13.923	**1.460**	5.579	**0.623**
	avg		1.539	**1.472**	7.747	**1.435**	3.986	**1.548**
avg			1.539	**1.479**	7.705	**1.416**	3.927	**1.455**

While the number of transactions per block can provide additional insight into network activity, Ethereum measures block capacity primarily in terms of gas usage rather than transaction count. Since different transactions may consume significantly different amounts of gas depending on the executed smart contract operations, gas used per block provides a more reliable indicator of network utilization and congestion. Therefore, our analysis focuses primarily on gas usage dynamics when evaluating the effectiveness of the proposed fee adjustment mechanism.

Across all fee mechanisms, scenarios with strategic users show slightly better performance than those with non-strategic users. This is because both EIP-1559 and our proposed method are designed to account for users who adjust their transaction submissions based on market conditions.

Our DRL-based method demonstrates significantly superior performance when transaction valuations are narrowly distributed - that is, when many users assign similar valuations to their transactions. The average standard deviation of gas used per block ($\sigma _y$) under the DRL method is approximately $1.4 \times 10^6$, compared to about $1.4 \times 10^7$ under EIP-1559 - a tenfold reduction. The rolling standard deviation of the base fee ($\sigma _{b,\text {rolling}}$) also shows a reduction of more than 25 times. Unlike EIP-1559, which relies solely on gas used per block to measure user demand, our method leverages state information through reinforcement learning, allowing for a more nuanced analysis of demand and more precise base fee adjustments.

When users’ transaction valuations are narrowly distributed, EIP-1559 struggles and becomes unstable across all scenarios, whereas our DRL-based model consistently maintains gas used per block close to the target. Figure 3 illustrates gas used per block under each methodology with strategic users in scenario 1, where $\lambda _t$ varies, and user transaction valuations follow uniform distributions over *U*(10, 100) and *U*(80, 90), respectively. When the valuation distribution is *U*(80, 90), EIP-1559 fails to function properly, while our proposed method maintains gas usage near the target through effective base fee adjustments. When the valuation range is broader, such as *U*(10, 100), EIP-1559 performs relatively better. However, our method still exhibits lower overall volatility in gas used per block and returns more quickly to the target gas level, especially during demand spikes. This trend is consistently observed across other scenarios, with graphical results presented in Supplementary materials.

### Effect of maximum change rate

Although Ethereum’s current EIP-1559 mechanism limits the base-fee adjustment rate to approximately 12.5%, we also consider a larger value of *d* to examine whether a wider control range can improve responsiveness under abrupt demand changes. To assess the impact of the maximum base-fee change rate on the DRL model’s performance, we compare settings with $d=0.2$ and the Ethereum-compatible value $d=0.125$, the latter reflecting the current protocol constraint.

Table 3 presents the DRL model’s performance across different *d* and with varying mempool information. When the user transaction valuation follows a uniform distribution over (10, 100), the DRL model with the maximum change rate of 0.2 performs better. However, when the valuation range is narrower, the model with the maximum change rate of 0.125 yields better results. A smaller *d* can prevent abrupt base fee changes and allows for more fine-grained adjustments when user valuations are closely clustered, but it can also slow the model’s response to sudden demand shifts.

**Table 3:** Comparison of DRL configurations with different maximum change rate of base fee (0.125,0.2) and state information ($\textbf{s}^2_t, \textbf{s}^2_t$).

User	Valuation	$\mu _y$ ($\times 10^7$)			$\sigma _y$ ($\times 10^6$)			$\sigma _{b,rolling}$		
		DRL(0.2, $\textbf{s}^2_t$)	DRL(0.125, $\textbf{s}^2_t$)	DRL(0.2, $\textbf{s}^1_t$)	DRL(0.2, $\textbf{s}^2_t$)	DRL(0.125, $\textbf{s}^2_t$)	DRL(0.2, $\textbf{s}^1_t$)	DRL(0.2, $\textbf{s}^2_t$)	DRL(0.125, $\textbf{s}^2_t$)	DRL(0.2, $\textbf{s}^1_t$)
Strategic	*U*(10, 100)	1.491	1.496	1.518	1.381	1.425	1.671	2.423	2.331	2.290
	*U*(80, 90)	1.481	1.346	1.749	1.413	1.353	2.298	0.302	0.277	0.333
nostrat	*U*(10, 100)	1.484	1.541	1.511	1.410	1.457	2.029	2.473	2.613	2.629
	*U*(80, 90)	1.460	1.367	1.595	1.460	1.367	1.837	0.623	0.284	0.246

**Fig. 4:**
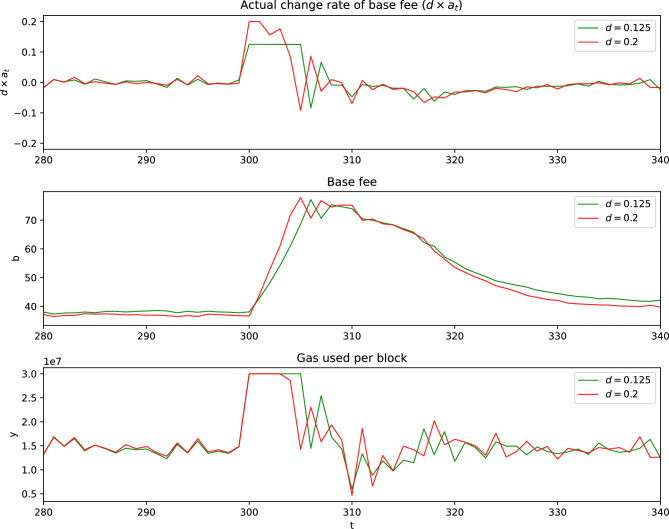
Comparison of DRL models with different maximum change rates of base fee in scenario 4 with strategic users and a valuation distribution of *U*(10, 100).

Figure 4 illustrates how the DRL models with different *d* react to a sudden demand spike in scenario 4. The model with the maximum change rate of 0.2 responds quickly to sudden demand spikes by rapidly increasing the base fee to the maximum change rate 0.2, causing gas used per block to quickly return to the target. In contrast, the model with the maximum change rate of 0.125 is slower in adjusting the base fee due to the smaller *d*, resulting in a slower return to the target gas. These experiments demonstrate that tuning the maximum change rate, *d*, helps the agent balance responsiveness and stability.

Unlike the rule-based update in EIP-1559, where increasing the maximum change rate directly leads to larger deterministic adjustments, the DRL-based approach learns a context-dependent action $a_t$. Even when the allowable range of *d* is larger, the learned policy can adaptively adjust $a_t$ according to network conditions, preventing unnecessarily large changes in the base fee. Therefore, allowing a wider maximum change rate can improve responsiveness to demand spikes without necessarily causing excessive fee volatility.

### Effect of memory pool information

We evaluated the importance of including mempool information in the state representation. When the agent has access to mempool statistics-such as the number of newly pending transactions, the number of transactions remaining after block creation, and user bidding information-it can make more informed decisions, leading to improved performance.

Our reinforcement learning model uses two state representations. The first option, $\textbf{s}^1_t$, considers only the base fee $b_t$ and the gas used per block $y_t$. The second option, $\textbf{s}^2_t$, expands on this by incorporating mempool information, including the number of newly pending transactions ($N_{new,t}$), the number of pending transactions ($N_{pend,t}$), and data about user bids ($\overline{f^{new,t}_{tip}}$, $\overline{f^{pend,t}_{tip}}$, $\overline{f^{pend,t}_{max}}$). By integrating this additional information, the agent can adjust the base fee more accurately.

**Fig. 5:**
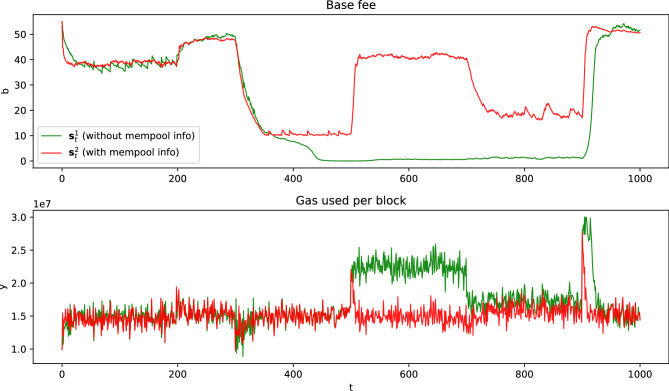
State feature comparison in scenario 1 with non-strategic users and a valuation distribution of *U*(10, 100).

Figure 5 shows the experimental results for scenario 1 with non-strategic users and a transaction valuation distribution of *U*(10, 100), comparing the two state options. The results demonstrate that including mempool information enables the model to quickly detect changes in user demand, leading to faster base fee adjustments and quicker stabilization of gas used per block to the target level. Additionally, Table 3 further confirms that including mempool information improves performance.

### Analysis of the relationship between excess gas and base fee

There has been ongoing discussion about potential issues with Ethereum’s current EIP-1559 transaction fee mechanism^34^, where the base fee may approach zero due to users optimizing their fees. To address this, Vitalik Buterin proposed the concept of *excess gas* and introduced a base fee calculation method based on the exponential function of excess gas^35^, which was recently implemented in EIP-4844 to determine the blob base fee^36^.

Excess gas $E_t$ is recursively calculated with each block as:

$$\begin{aligned} E_t = \max (E_{t-1} + y_t - y_{\text {target}}, 0) \end{aligned}$$

where $y_t$ is the gas used in the *t*-th block, and $y_{\text {target}}$ is the target gas. Excess gas $E_t$ quantifies the cumulative surplus gas used beyond the target, and the base fee is determined using the formula $b_t = A \cdot \exp {\left( \frac{E_{t-1}}{m} \right) }$, where *A* is a constant, effectively eliminating base fee path dependency^35^.

**Fig. 6:**
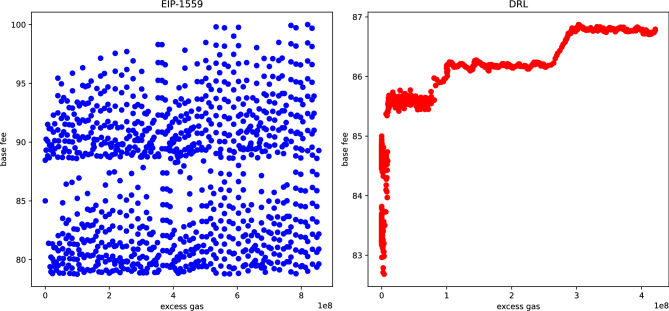
Comparison of excess gas and base fee between EIP-1559 and the proposed method in scenario 2 with strategic users and a valuation distribution of *U*(80, 90).

EIP-1559 effectively captures this exponential relationship when transaction valuations span a wide range. However, when transaction valuations are narrowly distributed, the base fee mechanism breaks down, and the expected relationship no longer holds. Figure 6 compares the relationship between excess gas and base fee under EIP-1559 and our proposed method in scenario 2, where user transaction valuations follow a uniform distribution on (80, 90) and strategic users are considered.

Unlike EIP-1559, our proposed method shows a positive correlation between excess gas and base fee, even though it does not explicitly assume path invariance. In future work, the model could be extended to incorporate path invariance within the reinforcement learning framework. For example, this property could be encouraged through reward design that penalizes path-dependent fee trajectories or through state representations that explicitly include cumulative excess gas dynamics. Such extensions would allow the learned policy to better capture the theoretically desirable relationship between excess gas and base fee.

## Conclusions

We introduced a novel DRL-based transaction fee mechanism for Ethereum that provides precise and adaptive base fee adjustments. Our approach effectively reduces variance in gas usage and transaction fees while enhancing responsiveness to sudden demand fluctuations, outperforming the existing EIP-1559 mechanism. Our findings demonstrate that the proposed DRL approach can be extended to more complex scenarios by adjusting key parameters such as the maximum change rate and reinforcement learning settings.

Future work will explore several directions to further improve the proposed framework. One direction is to incorporate additional economic factors observed in real blockchain environments. In particular, future studies may incorporate miners’ incentives related to MEV and extend the model to systems with multiple resources, such as the blob base fee introduced in EIP-4844^37–40^, by expanding the dimensionality of the actor network’s output. Another important extension is to incorporate more realistic priority-fee dynamics, which play a significant role in transaction inclusion in real Ethereum networks. Future research may also explore alternative reward formulations that explicitly incorporate additional economic objectives, such as transaction fee stability or user cost efficiency.

From a methodological perspective, another promising direction is to allow the adjustment parameter *d* to be dynamically learned rather than treated as a fixed hyperparameter, for example through meta-learning or hierarchical reinforcement learning frameworks. Future research could also investigate simplified adaptive or rule-based fee adjustment mechanisms that utilize richer state information and systematically compare their performance with the proposed learning-based approach.

In addition, validating the proposed mechanism in real-world blockchain environments remains an important direction for future research. By providing a more adaptive and resilient fee adjustment mechanism, our work contributes to the ongoing effort to optimize blockchain performance and user satisfaction.

## Supplementary Information

Supplementary Information.

## Data Availability

All data generated during the experiments are available from the corresponding author upon reasonable request.
