# Supplementary material for: A deep reinforcement learning approach for dynamic transaction fee adjustment in Ethereum
Source: Sci Rep. 2026 Mar 31;16:15600. doi: 10.1038/s41598-026-46368-2 (PMC13187429; doi:10.1038/s41598-026-46368-2)
Supplement: Supplementary file 1 — Supplementary Information. [file 41598_2026_46368_MOESM1_ESM.pdf]

A Graphical Results for Scenarios 2–5

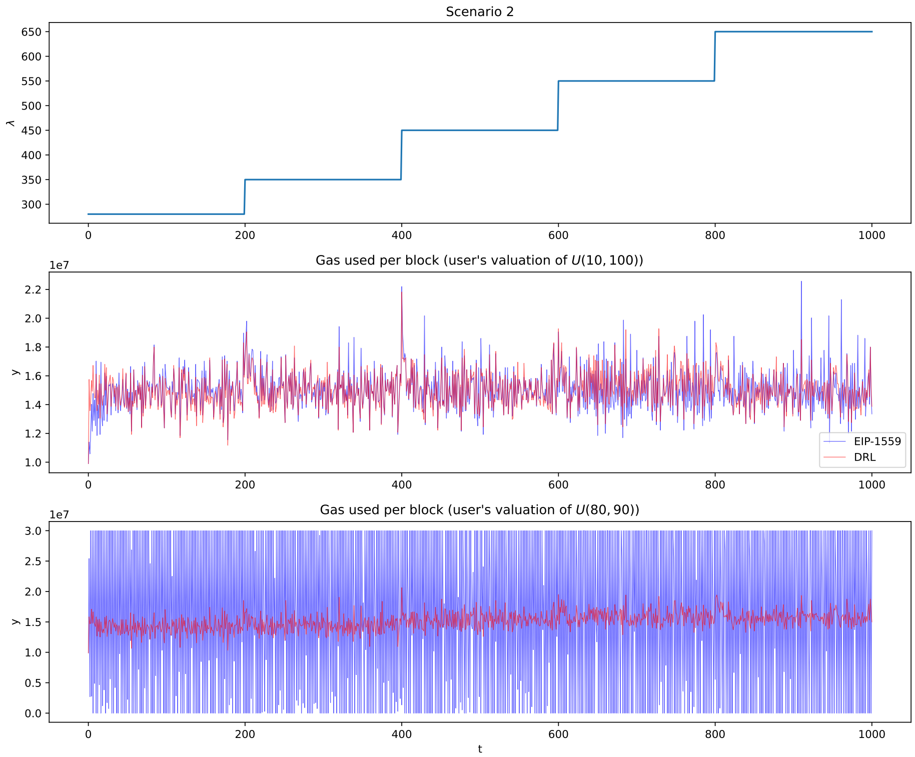

Figure 1. Gas used per block in scenario 2 with strategic users and valuation distributions of $U(10,100)$ and $U(80,90)$.

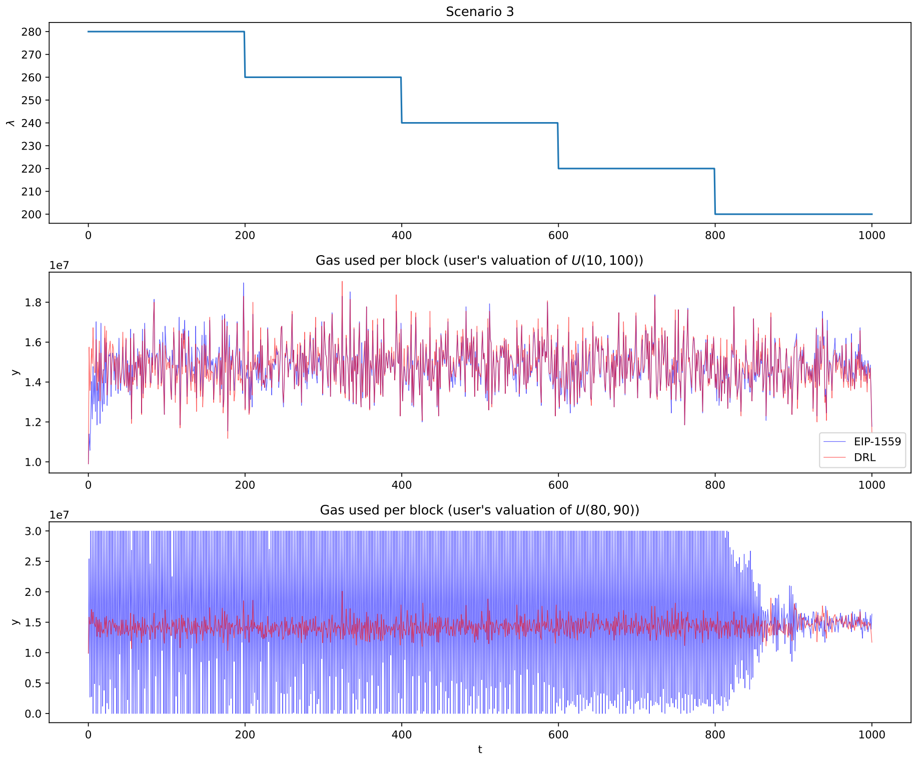

Figure 2. Gas used per block in scenario 3 with strategic users and valuation distributions of $U(10,100)$ and $U(80,90)$.

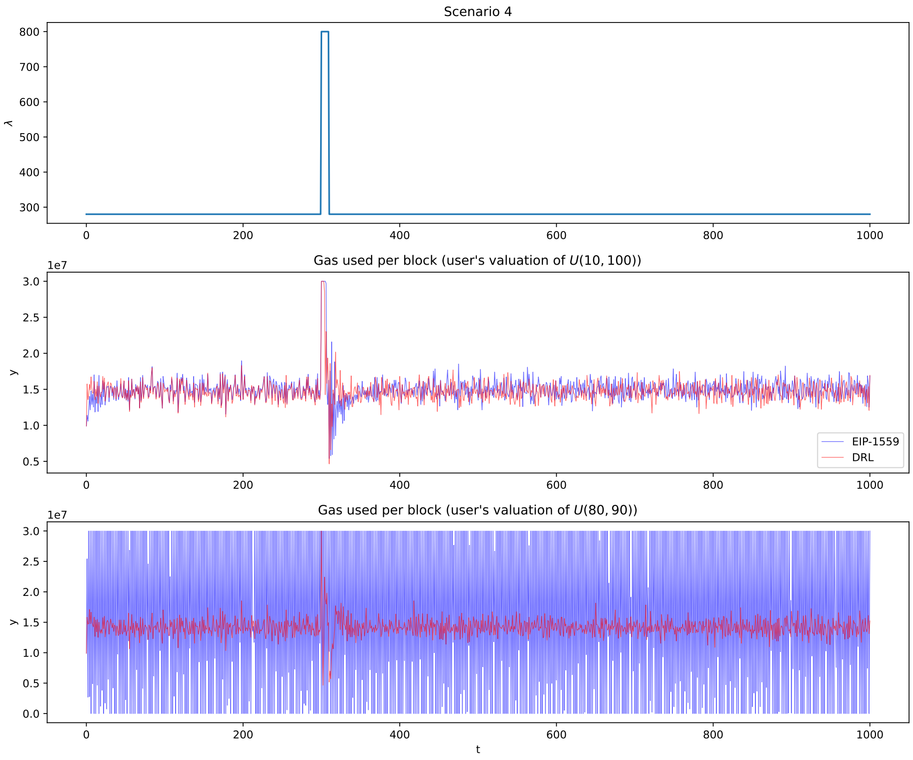

Figure 3. Gas used per block in scenario 4 with strategic users and valuation distributions of $U(10,100)$ and $U(80,90)$.

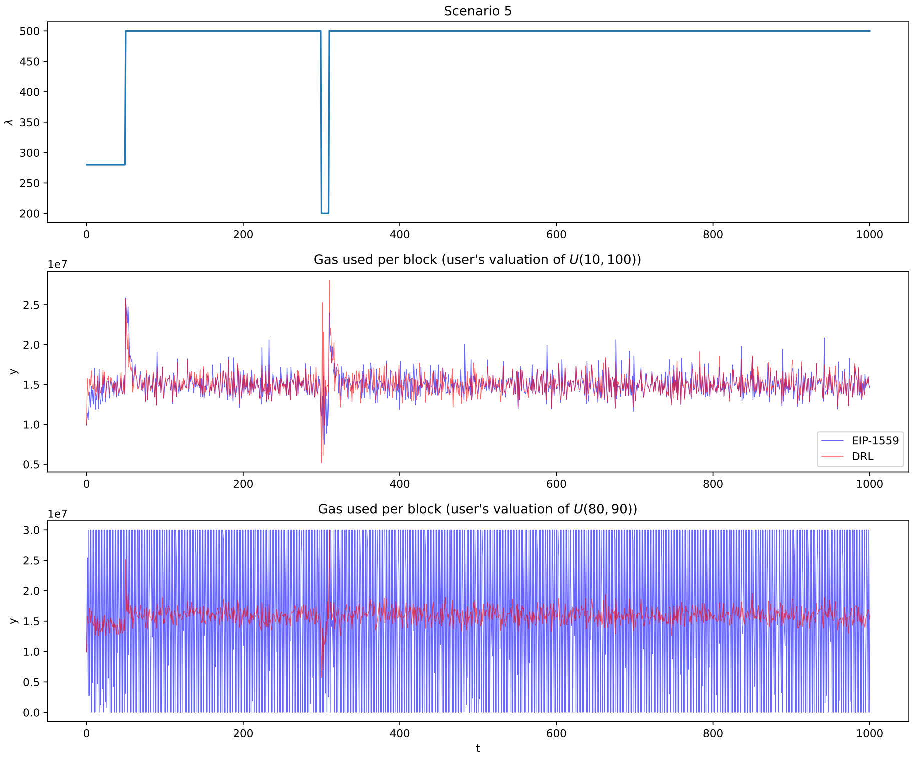

Figure 4. Gas used per block in scenario 5 with strategic users and valuation distributions of $U(10,100)$ and $U(80,90)$.
